# Supplementary figures and images for: BDNF signaling in correlation-dependent structural plasticity in the developing visual system
Source: PLoS Biol. 2023 Apr 3;21(4):e3002070. doi: 10.1371/journal.pbio.3002070 (PMC10101647; doi:10.1371/journal.pbio.3002070)

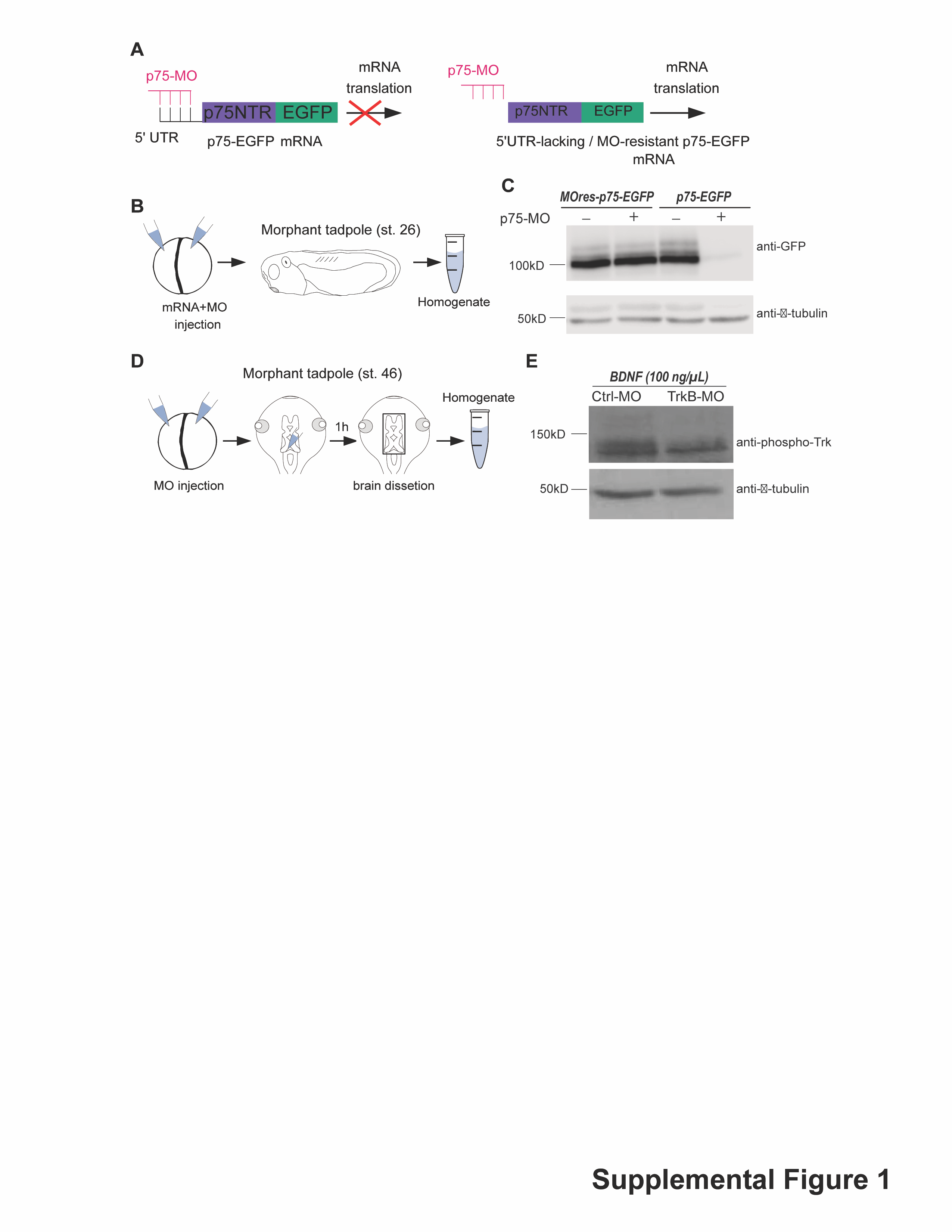

Supplement: S1 Fig — (A) Schematic of the mRNA constructs encoding p75NTR-EGFP fusion protein. Binding of p75-MO to the p75NTR-5′UTR-containing mRNA (p75-EGFP mRNA; left) impedes mRNA translation, whereas the inability of p75-MO to bind the construct lacking the p75NTR 5′UTR (MO-resistant p75-EGFP mRNA; right) spares mRNA translation. (B) Schematic of mRNA construct and MO injection at 2-cell stage, raising injected embryos to stage 26 and preparation of whole-animal homogenates. (C) Western blot analysis of homogenates derived from animals injected with combination of p75-EGFP mRNA or MO-resistant p75-EGFP mRNA and p75-MO probed for EGFP and β-tubulin. (D) Schematic of MO injection at 2-cell stage, followed by intraventricular injection of BDNF in morphant tadpoles at stage 46, 1 h before brain homogenate preparation. (E) Western blot analysis of brain homogenates probed for phospho-Trk (p-Trk) and β-tubulin. Approximate size (kDa) is shown next to the bands. The original western blot images used to generate S1 Fig can be found in S1 Raw Images. (TIF) [file pbio.3002070.s001.tif]

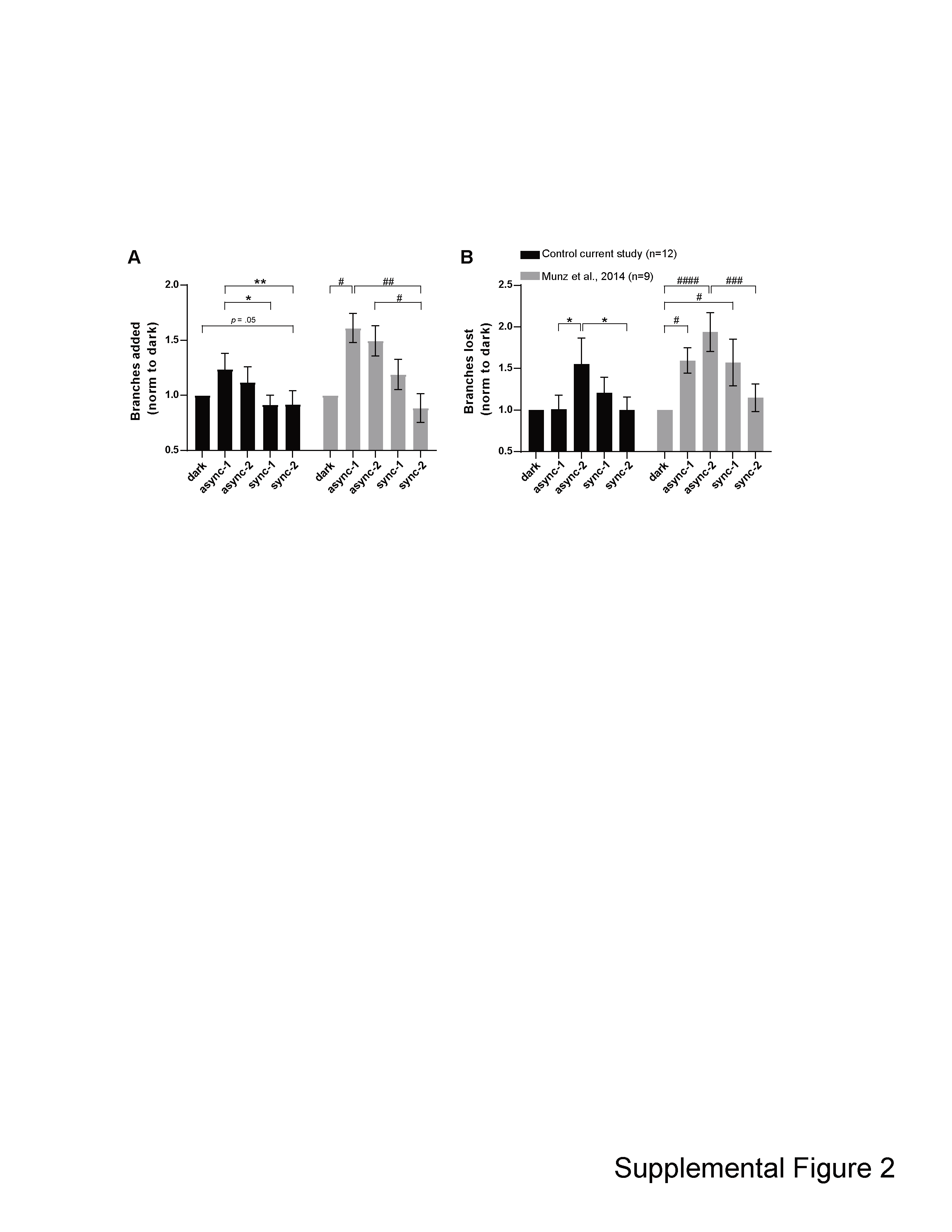

Supplement: S2 Fig — Branch (A) additions and (B) eliminations from the control group in the current study (black) plotted together with the control DAS group in Munz and colleagues, 2014 (Fig 4C and 4D), with the statistical significance shown as discovered in that study (gray). One-way nonparametric Friedman test was followed by multiple comparisons—uncorrected Dunn’s test (*p ≤ 0.05, **p ≤ 0.01), as the control group from the current study (black) is a replication study rather than a new discovery. Statistical significance as discovered by Munz and colleagues, 2014 (Fig 4C and 4D), # p ≤ 0.05, ## p ≤ 0.01, ### p ≤ 0.001, #### p ≤ 0.0001. Data represent mean ± SEM. Control from current study (n = 12), control from Munz and colleagues, 2014; DAS (n = 9). The data used to generate S2A and S2B Fig can be found in S7 Data. (TIF) [file pbio.3002070.s002.tif]

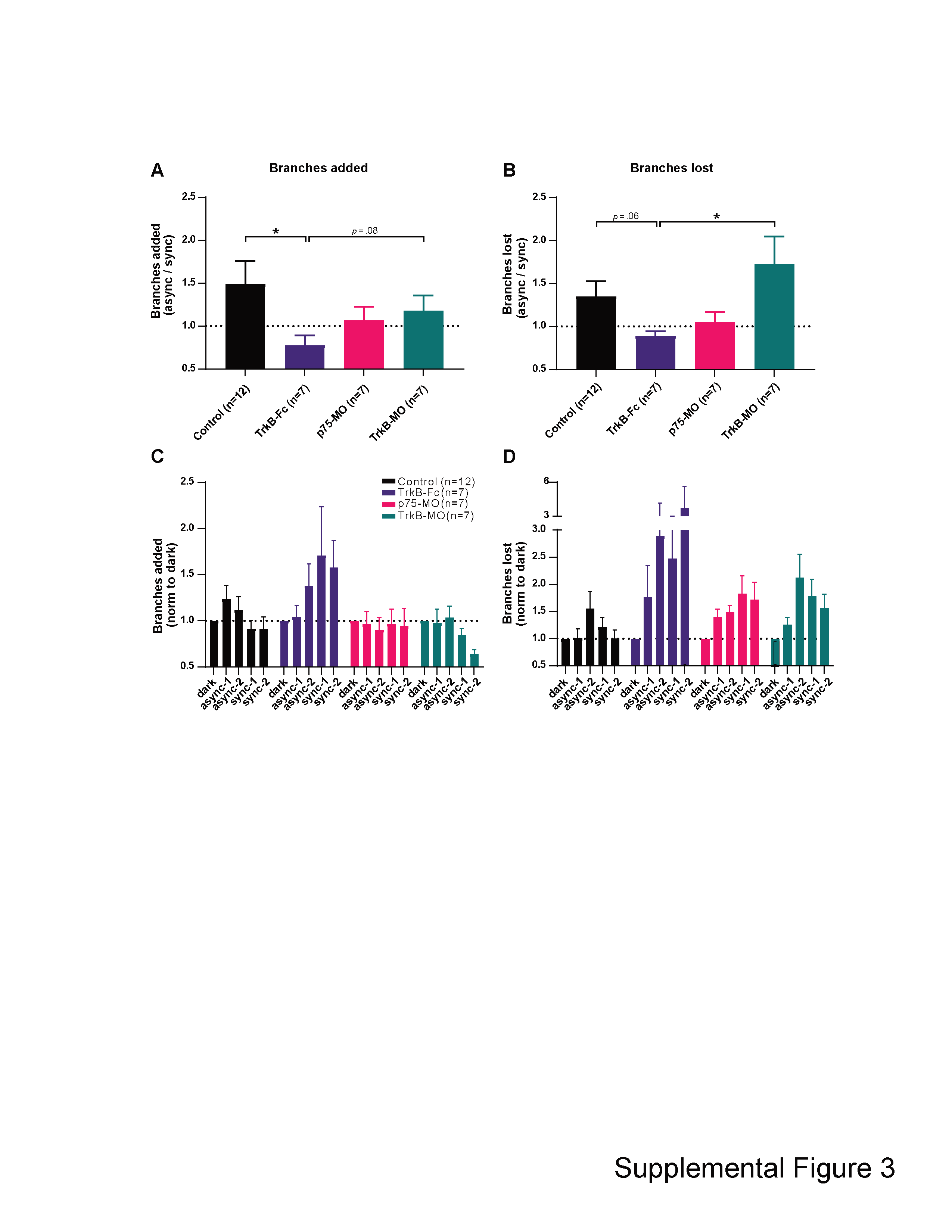

Supplement: S3 Fig — Branch (A) addition in the first hour of visual stimulation and (B) loss in the second half of visual stimulation are plotted as ratios of asynchronous to synchronous stimulation. One-way nonparametric Kruskal–Wallis for branches (A) added (p = 0.0556) and lost (p = 0.0346) test was followed up by pairwise post hoc tests corrected for multiple comparisons (BKY two-stage linear step-up procedure): *p ≤ 0.05. Branch (C) addition and (D) loss shown as average over 1 h for the MO-containing groups and 40 min average for TrkB-Fc. Data represent mean + SEM. Control (n = 12), TrkB-Fc (n = 7), p75-MO (n = 7), TrkB-MO (n = 7). The data used to generate S3A and S3B Fig can be found in S8 Data, S3C and S3D Fig in S9 Data. (TIF) [file pbio.3002070.s003.tif]

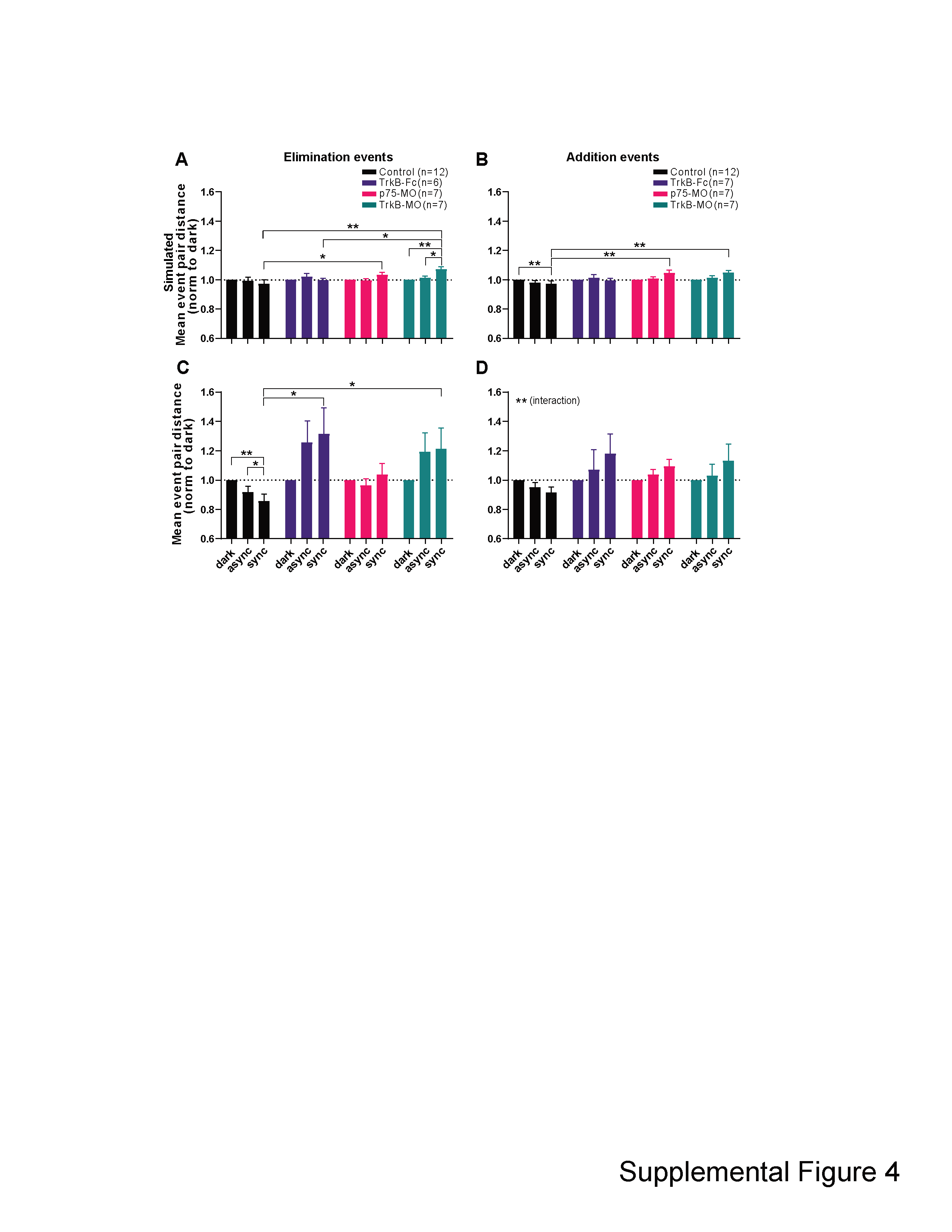

Supplement: S4 Fig — Mean pair (C) elimination and (D) addition and mean simulated (A) elimination and (B) addition event pair distances in DAS (1 h, 1.5 h, 1.5 h) derived by redistribution of events at random within the reconstructed arbor. Significant interaction in the two-way mixed design model: (A–C) p < 0.001; (D) p = 0.00866. Pairwise post hoc tests corrected for multiple comparisons (BKY two-stage linear step-up procedure): *p ≤ 0.05, **p ≤ 0.01. Data represent mean + SEM. (A–D) Control (n = 12), p75-MO (n = 7), TrkB-MO (n = 7), (A and C) TrkB-Fc (n = 6), (B and D) TrkB-Fc (n = 7). The data used to generate S4 Fig can be found in S10 Data. (TIF) [file pbio.3002070.s004.tif]

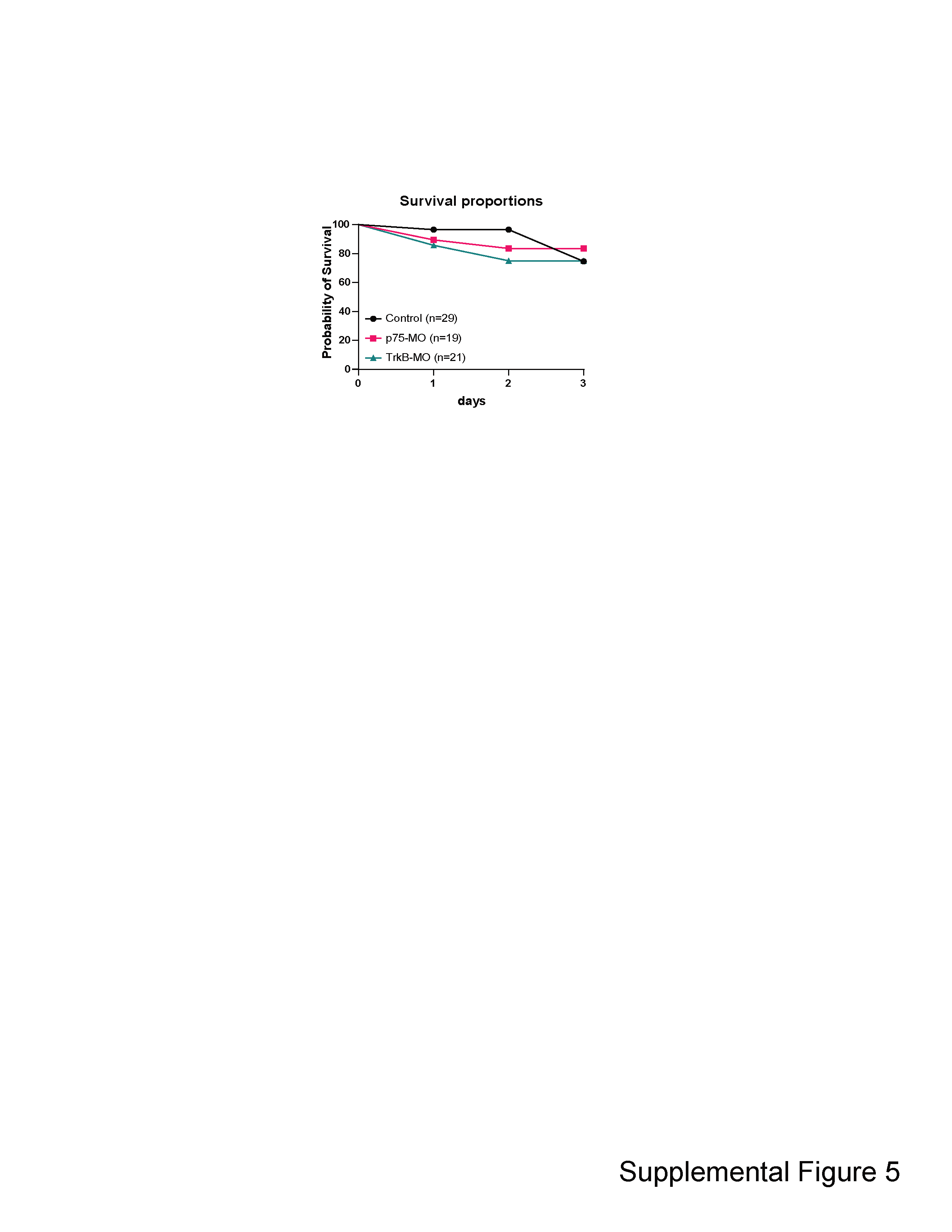

Supplement: S5 Fig — Survival proportions of contralaterally projecting RGC observed by daily 2-photon imaging in the optic tectum over 4 days. Day 0 corresponds to stage 45–46. CTRL-MO (n = 29), p75-MO (n = 19), TrkB-MO (n = 21). Log-rank test; χ2 = 0.3332; p > 0.1. The data used to generate S5 Fig can be found in S11 Data. (TIF) [file pbio.3002070.s005.tif]

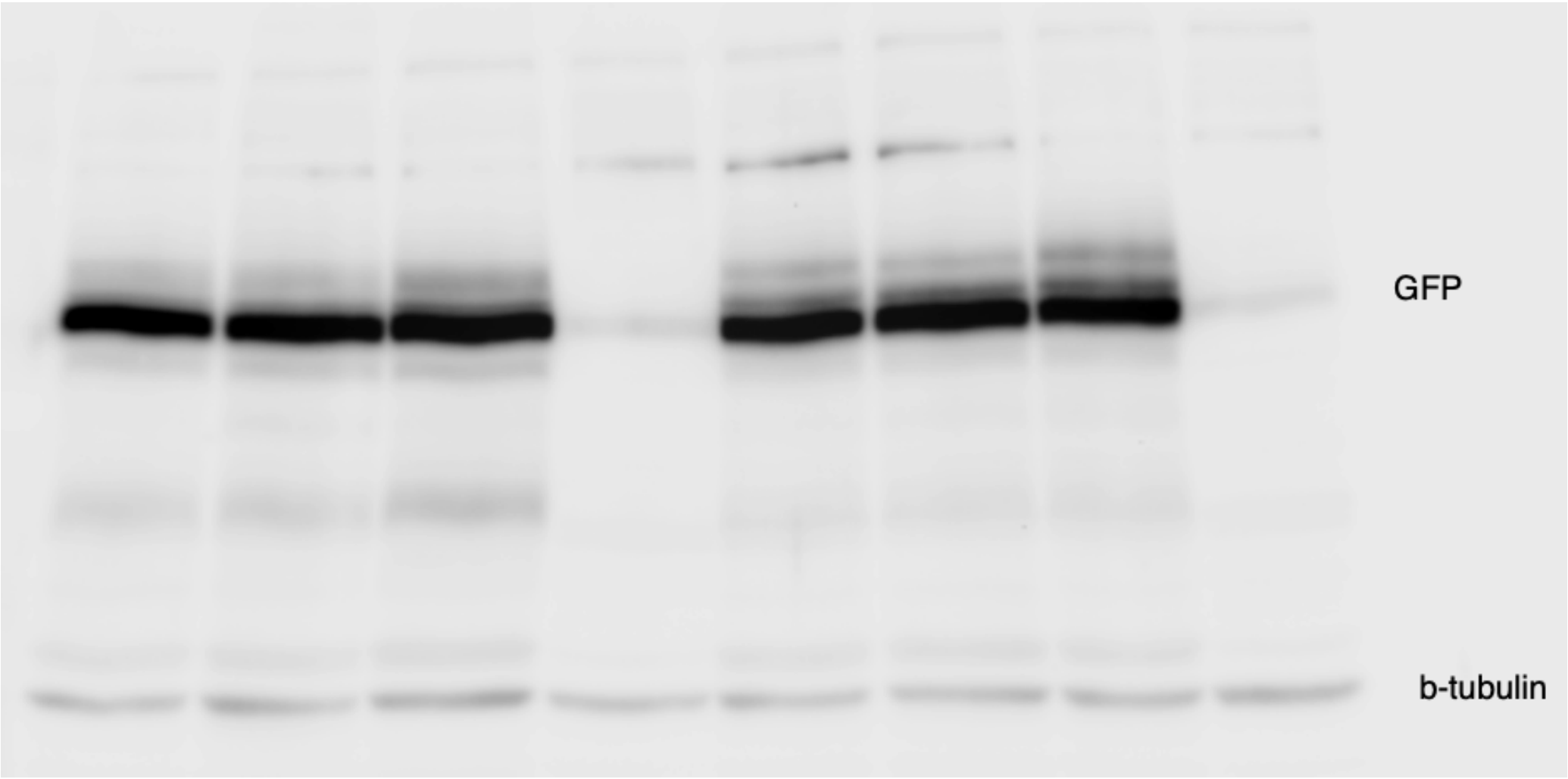

GFP

b-tubulin

02.02.12

problematic

15

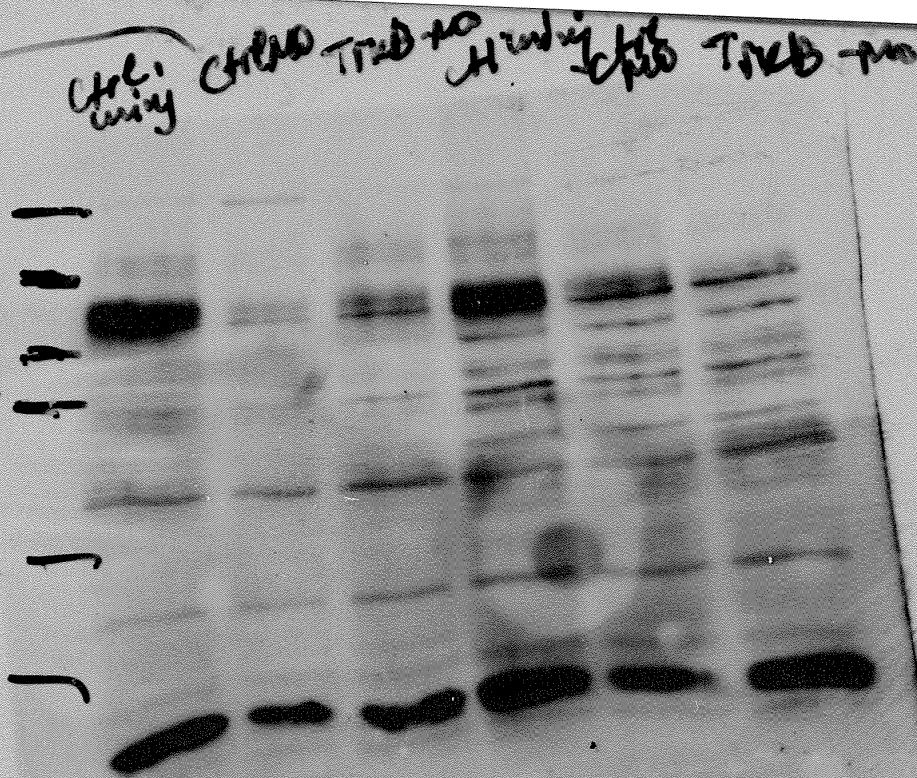

+ BDNF (100 ng/ml)

rap-T12B  
1:5000  
GAP 1:10,000

02.02.12

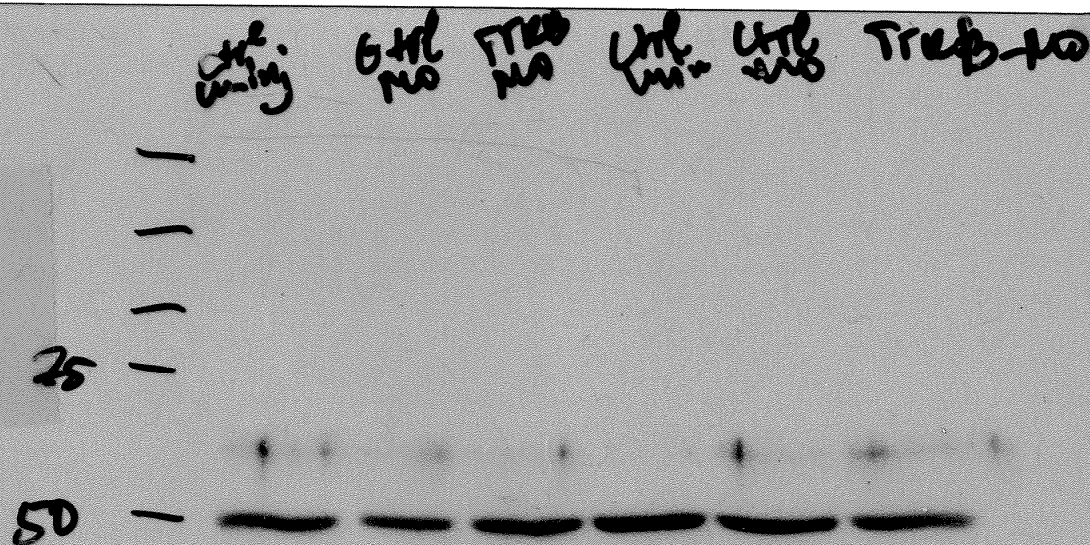

+ BDNF (100 ng/ml)

anti  $\alpha$ -tubulin

1: 20,000

Supplement: S1 Raw Images — Page 1: The 4 rightmost lanes of this blot, stained for EGFP and tubulin (loading control) were used to generate S1C Fig. Page 2: The 2 rightmost lanes of this blot probed for p-Trk were used to generate S1E Fig. Page 3: The same blot as on Page 2, stripped and probed for tubulin as a loading control for S1E Fig. (PDF) [file pbio.3002070.s018.pdf]
