# Supplementary material for: BDNF signaling in correlation-dependent structural plasticity in the developing visual system
Source: PLoS Biol. 2023 Apr 3;21(4):e3002070. doi: 10.1371/journal.pbio.3002070 (PMC10101647; doi:10.1371/journal.pbio.3002070)
Supplement: Attachment — Submitted filename: Reviews_20230115.docx [file pbio.3002070.s019.docx]

**Response to reviewers**

We have made a sincere effort to incorporate as many of the reviewers’ recommendations as possible. We thank them for their time and thoughtful comments.

**Reviewer #1:**

Kutsarova, Ruthazer and colleagues examined the role of BDNF, and its associated receptors TrkB and p75, on Hebbian and Stentian plasticity of ipsilateral retinal ganglion cell axons (RGC) during visual circuit development in albino Xenopus laevis tadpoles. The authors found that the presynaptic receptors, TrkB and p75, promote Stentian plasticity via RGC axon branch addition. Additionally, BDNF signaling had two significant roles in the Xenopus optic tectum: mediation of Hebbian plasticity via axon stabilization and suppression of local branch elimination. Although the paper describes relevant findings, the significance of the ipsilateral axons in albino Xenopus tadpoles needs to be further elaborated on as the reader is left wondering the exact importance of the findings. The paper also lacks the acknowledgements of significant literature relevant to this study, which can help put the new findings in context with the role for BDNF in Stentian plasticity. The clarity of the presented results could also be improved.

We have provided a more detailed justification and description of the power of using the ipsilateral axon to assess the effects of correlated activity on axonal structural plasticity and attempted to streamline the descriptions of the findings, which have been reanalyzed to help keep the paper more focused and clearer. We appreciate the reviewer’s comments about our insufficient reference to the relevant literature which fortunately for the Xenopus retinotectal system in particular consists of a number of high-quality studies that nicely synergize with our observations. We have made an effort to incorporate more references to these citations where appropriate

Specific comments:

There are several issues that the authors need to address in their presentation and discussion of the results, particularly with respect to previous evidence on the role of BDNF signaling on axon branch dynamics.

1. The authors should elaborate on whether ipsilateral RGC axons in albino Xenopus tadpoles at the stage examined do have a functional role in the circuit. This is important since the authors have found that only 20% of the tadpoles do have ipsilaterally projecting RGC axons (Rahman… & Ruthazer et al, 2020). Are these ipsilateral arbors only found in Albino tadpoles? Is this an error in pathfinding?

Sakaguchi and Murphey (1985) first reported these rare ipsilaterally projecting retinotectal axons and estimated that they constituted about 0.1% of the total population at these early stages. Nakagawa and colleagues (2000, Fig. 6, table 2), who studied the emergence of a significant ipsilateral projection during metamorphosis as the eyes move to more frontal positions, reported that there is only a modest proportion (~45%) of wildtype pigmented tadpoles at stage 41 that exhibit 1-5 uncrossed RGC axons. This is similar to what we reported previously in albinos (39%, Munz et al., (2014), Fig 1C). We found that a single axon is observed in about 20% of animals and even fewer animals have more than one ipsi axon. Thus, we strongly believe that the ipsi RGCs in the Xenopus tadpoles represent a serendipitous pathfinding error that is simply absent in most animals independent of pigmentation. However, our group has previously shown (Munz et al, 2014, Fig. 2) that the ipsi RGCs are able to form functional synapses with the optic tectal neurons.
We have added the following sentences (lines 78-85) for clarification:

“*We took advantage of the existence of ectopic ipsilaterally projecting (ipsi) RGC axons in albino Xenopus laevis tadpoles, which typically consist of just one or two axons and which spontaneously occur in fewer than half of all albino and wildtype animals* [*(Sakaguchi and Murphey, 1985; Nakagawa et al. 2000; Munz et al. 2014*](https://paperpile.com/c/hkGYUt/Enkn+0izs)*). Since these ipsi RGC axons are not observed in all tadpoles, it is more likely that they are a result of rare errors in pathfinding, rather than constituting a functionally specialized class of RGCs.”*

2. The authors examined "branch dynamics (addition and elimination of branches), but in many instances the branches measured are very small (1.5 micron) suggesting that these may be transient filopodia. How can one distinguish true branches vs filopodia? Do these filiopodia make active synapses?

Indeed, we have included filopodia in our analyses. The 1.5µm criterion is based on optical rather than biological properties, as it constitutes the smallest process, we can unambiguously distinguish from axonal swellings in 2P images. We believe that it is difficult to accurately disentangle filopodia from branches simply by length as we regularly observe short but persistent branches, as well as longer highly dynamic filopodia. Ultimately, we suspect (based on intuition rather than solid evidence) that many, if not all, filopodia have the potential to differentiate into branches if the conditions are right. Indeed, one could argue that “stabilization” may at least occasionally represent the conversion of an actin-based filopodium into a microtubule-bearing branch capable of supporting a mature synaptic contact. We therefore analyzed our axons for the paper without bias to not exclude such a possibility.

Out of curiosity, we recalculated our branch dynamics analyses with a cut-off of 5 µm (used previously by Cohen-Cory and Fraser, 1995) to generate the reviewer figure (see below arranged analogously to Figures 1F and G in the manuscript) in an attempt to exclude filopodia (“spikes”) from analysis. As you can see these plots largely preserved the effects, we reported in the manuscript using a 1.5µm cut-off. (Axons with no baseline addition or elimination events of branches longer than 5 µm in darkness were omitted from further analyses and thus the number of cells plotted on this figure differs from Fig.1 in the manuscript.)

We did not perform synaptic labeling in the ipsi axons and could not directly correlate the dynamic growth to the presence of synapses. However, synapses are active sites of filopodial sprouting (Alsina et al.,2001; Meyer and Smith, 2006; Ruthazer et al., 2006) and thus including filopodial growth is still quite informative for the synaptic status of the axon. Moreover, Munz et al. (2014) showed that the dynamic growth changes during asynchronous and synchronous visual stimulation in the same paradigm (including branches >1.5 microns) are correlated with synaptic strength changes (in other animals stimulated the same way). We thus have opted not to exclude these shorter processes from our analyses.

3. Treatment to observe effects on ipsilateral RGC axons must have involved transfecting most of the retinal neurons in one eye to then label the single RGC that projects ipsilaterally in that minority of the tadpoles (20%). This then means that the retinal circuit overall in one eye is affected by the manipulation. This is important to discuss or evaluate since both TrkB and p75 signaling have been reported to affect RGC development and differentiation locally within the retina. For example, Hutson and Bothwell 2001 have shown that interfering with p75 signaling affects RGC survival, Lom and Cohen-Cory 1999 and Lom et al 2002 have shown that BDNF affects RGC dendritic differentiation and therefore most likely RGC function locally within the retinal circuit. How can then those significant changes locally within the manipulated retina affect those sole ipsilaterally projecting axons?

Regarding RGC survival, Hutson and Bothwell, 2001 showed that overexpression of p75NTR increases the survival rate of RGCs, an effect which is specific for the first wave of cell death (stage 37-38) but not to stage 41. The morpholino electroporation in our study occurs at stage 40-42 and the imaging experiments occur at stage 46-48 (daily imaging) and at stage 47-48 (dynamic imaging of ipsi axons), thus the first wave of RGC cell death should not be influenced by our protocol. It is, however, possible that later waves of cell death may have been impacted by our manipulation. To assess whether this was the case or not, we performed a survival analysis of contralaterally projecting RGCs electroporated with Control, p75NTR or TrkB morpholino antisense oligos (day 0 corresponds to stage 45-46). We did not find any significant differences (added here and as Fig S5) in the survival curves of Control (electroporated with MO), p75-MO and TrkB-MO electroporated RGC axons (Log-rank test; χ^2^ = 0.3332; p>0.1).

As suggested by the reviewer, based on the findings of Lom and Cohen-Cory (1999) and Lom et al., (2002), it is indeed likely that a knock-down of the BDNF receptors in the retina would result in changes in dendritic RGC morphology. As BDNF affects RGC dendritic branching, knocking-down its receptors could lead to mistargeting of the dendritic arbor and thus altered direction selectivity, or changes in laminar ramification and thus hindered on/off selectivity. However, Lom et al. (2002) specifically addressed the question of whether retinal BDNF manipulation might affect RGC axonal morphology in the tectum and found that although there was a strong impact on dendritic arborization, no changes could be observed in the RGC axons. This is an important point which has now been included in the discussion in lines 315-320

4. The authors need to discuss their results in relation to published studies that have shown that TrkB signaling (dominant negative downregulation in single Xenopus RGCs through specific truncated TrkB expression; Marshak et al 2007) interferes with the dynamic branching of RGC axons (addition and elimination of branches), alters growth cone morphologies, and importantly affects synapse formation

We agree that it is important to present our work in the context of these important previous findings. We have therefore updated the discussion to include this contextualization in lines 363-369.

5. Why are changes in branch dynamics are only observed by four days after Morpholino injection (Figure 3), when presumably expression of proteins begins to recover?

It is generally true that knock-down with morpholinos injected into the early embryo is considered to be most efficient for the first 4 days postfertilization in zebrafish. However, for example, in Figure S1E, we show that after injection of TrkB-MO at the 2-cell stage, we observe a decrease in phosphorylation of Trk about a week later at stage 46. Furthermore, in most of our experiments the MOs were electroporated into postmitotic neurons that had already begun expressing p75NTR and TrkB. We have found that transmembrane proteins can take longer to fully deplete than cytosolic proteins, perhaps due to slower turnover in the membrane.

6. In general, all graphic data is presented in a manner that is difficult to understand, especially since the bars are noisy and the legends to figures lack detail explanation of what is presented in the graphs.

We thank the reviewer for this comment. We have changed the graphs to display the data as mean ± s.e.m instead of individual data points or interquartile range. This makes the trends much easier to see and understand. In order to maintain full transparency, the data used in the graphs will be uploaded as spreadsheets in the supporting data. We have updated the statistics table (supplemental table 1) and tried to indicate the statistical significance more clearly in the figures.

7. Lines 247 - 254. Here, the authors need to acknowledge published work that specifically showed a role for BDNF in axon branch stabilization in Xenopus (Hu et al, 2005). Whether those effects could be pre- or postsynaptic was also addressed in another paper studying Xenopus RGC axons published in 2006 (Sanchez et al, Development 2006).

We have now discussed our findings in the context of these studies which are cited throughout the paper, and in particularly on lines 310-315. Thank you for this valuable comment.

8. Lines 282-292. The logic in this portion of the discussion somewhat difficult to follow.

We have rewritten the paper extensively and we hope that this part of the discussion is clearer now.

9. Lines 292-300. Here, the authors can also refer to Hu et al, Development 2005 to discuss how their new results are consistent with findings on the role of BDNF and activity on RGC axon branch stabilization in Xenopus.

We have now discussed our findings in the context of these very relevant studies.

10. The authors should provide a more detailed explanation for the model shown in Figure 4G.

We have provided a detailed explanation of the model in the figure legend of the new Figure 5.

Reviewer #2: Review of Kutsarova et al., PLoS Biology

"BDNF signaling in Hebbian and Stentian structural plasticity in the developing visual system"

Summary:

The authors applied a combination of dual-eye optical stimulation, live-cell imaging, morpholino-based receptor knockdowns, and competitive BDNF sequestration to study the role of BDNF signaling in the development of RGC axons in the Xenopus tectum. The key findings are that 1) deletion of p75 and TrkB receptors from RGCs changes axon branching dynamics in response to synchronous/asynchronous RGC stimulation (presynaptic effects), 2) extracellular sequestration of BDNF by application of TrkB-fc protein fragments increases branch addition in axons of synchronized RGCs (postsynaptic effects), and 3) RGC expression of p75, but not TrkB, is required for axonal elaboration. The experiments are rigorous in their design and the data quality is high. This manuscript will be of general interest to researchers investigating mechanisms of neural development and will be of special interest to researchers studying the structure, function, and development of visual circuits as well as mechanisms of Hebbian/Stentian plasticity. There are several concerns that should be addressed prior to publication: 1) the results of the current study do not appear to recapitulate results from a previous study from the same group, 2) the discussion of the different effects of p75 versus TrkB KD on axon development could be more clear, 3) the authors may overstate certain interpretations/conclusions - a more balanced discussion would be helpful, and 4) the presentation/discussion of statistically-significant findings is unclear in several places.

Overall:

This reader found it challenging to parse the relative contributions of p75 and TrkB signaling to axon remodeling and a more clear discussion would be helpful. The manuscript will also be improved by including a direct comparison of the current data with previous results from Munz et al. 2014. Analysis of additional biological replicates may help to address issues with variance and trends in the results.

This is a helpful suggestion. We have created a supplemental figure to directly compare Munz et al. (2014) with the current study. The discrepancies are in fact relatively minor, and the fundamental effects are clearly present in both, though effect size is definitely smaller in the current study.

Major concerns:

1)	The data presented in Figure 1 E-G measuring branch loss do not recapitulate previous results reported from the same group and this raises questions about the interpretation of these experiments on BDNF's role in branch elimination/stabilization. Munz et al. 2014 showed that in a dark/asynchronous/synchronous (DAS) stimulation paradigm, asynchronous stimulation increased branch loss almost two-fold compared with dynamics in the dark. Thereafter, synchronous stimulation did not affect branch loss. These effects were dependent on the stimulus order and DSA stimulation caused increased branch loss following both synchronous and asynchronous stimulation. In the current study, Figure 1 E-G presents the results of DAS stimulation and reports no differences in branch loss across the stimulation paradigm in control ipsiRGCs. In lines 130-134 the authors write: "Knock-down of p75NTR or TrkB in the ipsi RGC axon, as well as broad depletion of BDNF signaling, led to an overall

increase in the rates of axonal branch loss in response to visual stimulation compared to control ipsi axons (Figures 1E and 1G). Thus, under normal conditions, BDNF signaling can help stabilize axons against activity-induced axonal branch loss." However, this interpretation is based on the finding that branch loss is essentially unchanged in control ipsiRGCs across the DAS protocol in the current study. This raises several questions that should be addressed prior to publication:

a. What is the nature of the discrepancy between branch loss levels on control ipsiRGCs measured in the current study and those reported in Munz et al. 2014? If the authors compare their current control data set with the previous data from Munz et al 2014 do they find statistically significant differences in rates of branch addition/loss between control data sets across the two studies?

Upon careful examination of the rates of branch additions and losses over the whole ipsi imaging experiment, we saw that the data from this study recapitulates the dynamics of the branch addition increase and decrease in Munz et al. (2014), now shown side by side in Figure S2. For example, according to the Munz study, the highest increase in branch addition occurs within the first hour of asynchronous stimulation, whereas the highest increase in branch loss takes place during the second hour of asynchronous stimulation. We see very similar dynamics in the control data collected in the current study. In S2 Fig (reproduced above), the data are shown as 1h bins throughout the whole stimulation period.

The increase in both branch addition and loss during asynchronous stimulation appears smaller in this study, but the central effect and timing are identical. It is plausible that the small differences in experimental procedures over time have produced slightly different results. For example, the PDMS imaging chamber used here (and in Rahman et al. PNAS, 2020) to permit better stabilization of the tadpole for imaging during perfusion with oxygenated MBSH was not used in the Munz (2014) study.

b. If the authors perform additional imaging experiments can they replicate the findings of Munz et al 2014 for branch loss and, if so, what would this mean for the findings reported in other experiments with similar replicate numbers to the current control sample data?

We have added 3 more control ipsi axons to the data set (n=12). Two of them contain Control-MO and were imaged and traced blind to the experimental design, similar to the rest of the dataset in this study, but one of them was electroporated with EGFP only and the imaging experiment and arbor reconstruction were not performed blind to the experimental design. Below is a figure which shows the data from Figure 1F-G with and without the three newly added neurons compared to the data from Munz et al, 2014. The newly added neurons do not change the direction of any of the effects. Thus, although we agree that additional replicates in the rest of the conditions would have strengthened the conclusions, it is unlikely that they would change the direction of the findings. Due to the challenging nature of these experiments, the replicate number in the BDNF-manipulation group in this study is modest but still comparable to that in Munz et al., 2014 and Rahman et al. 2020.

Additionally, we interpret the lack of significant difference between asynchronous and synchronous stimulation in branch additions (Fig.1F) in TrkB-MO and p75-MO only in the context of the significant interaction of the two-way mixed design test. An additional supplemental figure in which the branch dynamics data are shown as ratios (Fig. S3A-B) of asynchronous to synchronous stimulation provides another way to assess the difference between asynchronous and synchronous stimulation across the groups.

c. The current study uses a dark/asynchronous/synchronous (DAS) stimulation protocol, which according to the Methods appears to be a fixed paradigm for all experiments. Previous work from the group (Munz et al. 2014) shows that branch remodeling dynamics depend on the stimulus order, DAS/DSA versus DSA/DAS. A discussion of how the stimulation history impacts branch dynamics and BDNF signaling would help the reader better understand and contextualize the results of the current study.

Of course, these experiments are too time consuming to include 4 experimental molecular manipulations and also double the conditions by adding a DSA series. But because this is an important point to consider when evaluating the results, we have now added two full paragraphs (lines 386-417) to the discussion on the influence of stimulation history on observed dynamic and long-term remodeling of the axon. We thank the reviewer for this suggestion.

d. The results presented in Figure 2 on branch loss data in control ipsiRGCs are difficult to interpret due to the aforementioned discrepancy between the current study and Munz et al., 2014. A comparison of the current data with those presented in Munz et al. 2014 would be helpful for understanding the lack of any effect on branch loss during the asynchronous stimulation epoch.

We have now provided the requested comparison as S2 Fig.

e. The data presented in Figure 1 show significant variance between replicates. The authors should comment on the sources of this variance and how this may impact the interpretation of their results. For example, in panel F the control data show that in four experiments asynchronous stimulation caused a reduction in branch addition while in four other experiments more branches were added. Overall, the cumulative data for Panel F show a statistically-significant increase in branch addition with asynchronous stimulation but the effect is being driven primarily by two animals with 2/2.5x increases in branch addition relative to the dark period. Similar outliers exist in the morpholino data and raise questions about how this variance contributes to the overall conclusions.

In *Xenopus laevis*, there is not a convenient way to use genetic access to specific classes of RGCs. Therefore, using retinal electroporation, we may label different classes of RGCs which exhibit varying behaviour in response to the stimulation. Selection is usually performed based on morphological features which correspond to a “healthy” RGCs, e.g. certain degree of arborization of the axon in the optic tectum, and certain level of basal branch dynamics. Since these experiments were performed blind to the experimental condition, and the experiments aimed at revealing differences in both arborization and dynamics, there was no preselection based on arborization or basal branch dynamics which may explain the increased variability in control neurons. In the two morpholino conditions variability may arise from a different level of knock-down achieved in the neurons of interest. Although we electroporated the same concentration of morpholino across all the experimental replicates, we do not have control over how much morpholino gets taken up by a particular neuron and thus over the level of knock-down of the proteins. For the purposes of this study, we have not excluded any axons based on their morphology or branch dynamics.
 Throughout this study, we performed non-parametric statistics only, which should avoid statistical significance being driven by a small number of “strong responders”. The increase in asynchronous stimulation in comparison to synchronous stimulation is now quite clear In the Control group.

f. Similar to the variance issue described in (e), there are trends in the data that may be clarified by analyzing additional biological replicates. In Figure 4E, it appears that p75-MO axons are not significantly smaller than controls and TrkB-MO axons are not significantly larger than controls, while p75-MO and TrkB-MO are significantly different from one another. Analysis of additional replicates may strengthen the conclusions in this section of the work.

We do agree that a large number of additional replicates could have strengthened the conclusions from the daily imaging. We prioritized acquiring additional replicates in the ipsi experiments. For all the figures, we have now also reported p-values of “trends” where p < 0.1 – the alpha of 0.05 for significance is quite arbitrary after all. The bottom line is that these ipsi RGC imaging experiments are exceptionally difficult, fraught with low yields (about 1 tadpole per 50 electroporated) for obtaining an animal with an imageable ipsi axon in the first place, then requiring that it be well co-labeled with both EGFP and the MO, and finally successfully maintaining the animal in a healthy state for about 6 hours of continuous live imaging at 10 min intervals. Finally, the painstaking manual reconstruction and dynamics analysis for over 30 time points per cell can take at least a week to perform per animal. As much as we would like to have a more highly powered dataset, there are practical limits in this case.

2)	The results from Figure 1 show that TrkB-MO and p75-MO manipulations lead to similar outcomes on branch dynamics of ipsilaterally projecting RGCs. In contrast, the results in Figures 3/4 show that p75-MO leads to reduced complexity of contralaterally projecting RGCs, while TrkB-MO has no effect. What is the interaction/compensation between these two pathways for BDNF signaling? Did the authors perform dual KD of both receptor types in any experiments? In lieu of new experiments, a more thorough discussion would help the reader better understand these results. Currently it is challenging for the reader to reconcile the similar effects shown in Figure 1 with the divergent effects shown in Figure 3/4. In lines 232-238, the authors write: "It is therefore plausible that the level of correlated neural activity among contra axons obscures the effects of Stentian plasticity for all but the most imprecisely targeted stray axons over days. The short-term dynamic imaging of ipsi axons, which allowed us to systematically alter the degree of correlation in RGC firing, unmasked a role for presynaptic TrkB in contributing to Stentian structural plasticity through an increase in the rate of new branch addition (Figures 1D and 1F)." This discussion is confusing. Given that short-term branch dynamics are similar (Figure 1), a clear and thorough discussion of the diverging results between p75 and TrkB in Figure 3/4 is needed. This confusion is captured in lines 303-304 where the authors write: "We propose that presynaptic p75NTR and signaling promote aspects of Stentian exploratory growth…" - exactly which aspects is unclear in the current discussion.

Some of the confusion came from an absence of sufficient supporting context for these sentences, but much also came from the very busy and frankly confusing figures in our original submission. The revised versions of the figures make it much easier to see what the main effects of our manipulations are and we have made a greater effort to contextualize the statements in the descriptions.

In essence the point is that the ipsi axon offers the possibility of imaging a single axon while its degree of activity correlation relative to its neighbors is completely under our control. This is something that is nearly impossible to do with a normal contralateral axon. We imagine that for a normal projection one small side branch of the arbor might land in territory where most of the other inputs come from a distant part of the retina, but most of the arbor projects among other inputs with very similar receptive fields. Thus, the effects of asynchronous activity on arbor growth are “masked” by the vast majority of synchronous interactions present.

Due to the laborious nature of these experiments, we unfortunately were not able to attempt to do a double knock-down for p75NTR and TrkB in the same cell.

3)	Lines 202-205 the authors may overstate the conclusion: "Our finding that p75NTR promotes the elongation of axonal branches specifically through the extension of the shortest filopodium-like branches (Figure 3G) further clarifies the role of p75NTR in RGC axonal exploratory growth." The current study relies on loss-of-function experiments so the results are more appropriately discussed as "X is necessary/required for Y". More specifically, the authors have not provided direct evidence that p75 promotes axon elongation- this would require overexpression experiments. The authors should take care throughout to not overstate their conclusions.

We have now taken care to address this issue in the text by restricting the word “promote” to describe our hypothetical working model rather than the direct experimental observations.

Minor concerns:

1)	Line 53: "evidence indicates" is vague and this sentence could be more helpful for the reader if it included a mention of the specific data or analytical approach: e.g. "previous in vivo imaging experiments…"

We thank the reviewer for this suggestion. The text has now been clarified.

2)	Lines 55-56: the transition between the discussion of Hebbian/Stentian plasticity and BDNF function is abrupt. If the authors can expand the text to have a logical segue from activity patterns to BDNF release, this would help maintain logical flow in the writing. The following sentence beginning in line 58: "BDNF can be synthesized and released in a constitutive or activity dependent manner from axonal terminals and dendrites…" helps to capture the idea that activity patterns/levels govern molecular signaling events and should perhaps be moved up in the text to immediately follow the Hebbian/Stentian discussion.

We hope that the introduction follows a clearer logic now.

3)	Line 103: typo "the entire the"

We thank the reviewer for pointing out this typo. We have now corrected it.

4)	The images in Figure 1A are confusing. If the MO-lissamine constructs are electroporated into the eye, why are there large fluorescent artifacts in the tectum (magenta signal right panel)?

Upon microglial engulfment fluorescence from labeled RGC axonal arbors can be observed in lysosomes of microglia in the optic tectum. The fluorescence of EGFP is markedly quenched with decreased pH typical of lysosomes, though microglia that have uptaken ph-resistant EGFP from axons can be visualized (Lim and Ruthazer, 2021). On the other hand, lissamine fluoresces more stably across wider ranges of pH and thus the “artifact” observed in Fig1A is lissamine-positive microglial cell.

5)	Lines 156-159: The authors write: "Extracellular depletion of BDNF with TrkB-Fc, or knockdown of p75NTR or TrkB in ipsi axons, resulted in a redistribution of branch elimination events, no longer favoring the event proximity normally seen with synchronous stimulation (Figure 2E)." Were the results of the p75 KD and TrkB KD statistically significant? If yes, this should be indicated in the Figure and accompanying text.

There is a significant interaction of the overall 2-way mixed design model (across the 4 groups) and a significant difference between darkness and synchronous stimulation in Control cells that is no longer present in p75-MO and TrkB-MO. We agree that a direct comparison between synchronous stimulation in control axons and TrkB-MO or p75-MO axons would have strengthened this conclusion. However, the loss of the decrease that is observed in Control axons is meaningful in the context of a significant interaction. Nonetheless, we have opted for an interpretation focused on BDNF signaling (based on the strong effect of TrkB-Fc manipulation) rather than attributing the effects to any of the presynaptic receptors.

6)	Line 165: "Axon arbor elaboration over days relies on p75NTR expression in the RGCs" reads somewhat awkwardly. Consider revising this heading title for clarity. Specifically "the RGCs" stood out as awkward and this phrasing appears elsewhere in the text.

We have changed these instances now.

7)	Figure 2 requires a scalebar to accompany the reconstructed axons. Figure 1C also requires a scalebar to accompany the reconstructed axons.

We have now included scale bars in these figures.

8)	The Euclidian measurement approach used to produce the results in Figure 2 precludes any investigation of local interactions along individual branches. Have the authors considered a measurement of branch dynamics using an on-path analysis that relates events by their proximity along individual branches? This would be a more biologically-relevant and informative measurement.

We computed the intracellular path of a pair of events by using the hierarchical dependencies of the nodes from the traced skeletons to calculate the shortest path between two event nodes (either elimination or addition) in a graph object. We then calculated the intracellular distances of all pairs of events and computed their mean analogously to Figure 2D. The data plotted below are comparable to the data in Fig. 2E-F, i.e. are corrected for morphology-driven event pair distances, calculated by Monte Carlo randomization of the events occurring within a stimulation period. Statistically significant interaction in the two-way mixed design F-test was observed for the (A) elimination events (F(6,56)=2.3637; p=0.041795) but not for (B) addition events (F(6,58)=1.13811; p>0.1). The main effect in control neurons, (i.e. a decrease of the elimination event pair distance observed when calculating the Euclidean distances) is not significantly different when calculating the intracellular paths, although appears to have the same direction of changes. Similarly, sequestering BDNF extracellularly with TrkB-Fc does not lead to an increase in elimination event intracellular pair distance. This may reflect that the “clustering” of branch eliminations during synchronous stimulation is primarily driven by extracellular signals, one of which appears to be BDNF.

9)	The Figure 3 legend should explicitly state that experiments are performed in contralaterally projecting RGCs.

The Figure 3 and 4 titles now include the words “contralaterally projecting RGCs”

10)	The statistics in Figure 4 are unclear. The authors write "with TrkB-MO arbors expanding more rapidly over 4 days to occupy a greater volume in the optic tectum compared to p75-MO axons (Figure 4D-E)" (lines 190-192) and later "Indeed, we observed that TrkB knock-down in RGCs led to a notably enlarged axonal arbor span (Figure 4)." (lines 297-298). "Notably larger" is vague- compared to what exactly (p75-MO or controls)? Is this result statistically significant comparing TrkB-MO to controls? Figures 4 D/E should have very clear presentations of the statistical comparisons to help the reader understand the results.

The interaction of the 2-way mixed design model is significantly different in Figure 4D (now marked with asterisks and the word interaction in panel D), which suggest a difference in the rate of arbor field expansion. In Figure 4E, we show a significant increase between the arbor field expansion index of TrkB-MO and p75-MO. We have added p<0.1 as trends which may be useful to disentangle where the difference between the two manipulation groups comes from, but we do not use the trends for interpretation in the text. We have now changed the text to clearly state that the difference in expansion index is between TrkB-MO and p75-MO.

11)	Are the effects of p75-MO shown in Figures 3/4 related to TrkA-mediated axon maintenance? A discussion of Singh et al. 2008 "Developmental axon pruning mediated by BDNF-p75NTR-dependent axon degeneration" would be helpful.

We now discuss this study in the context of activity-dependent competition, synapse and arbor stabilization and the difference between these processes between the PNS and the CNS. In *Xenopus laevis* tadpoles it has been shown that injection of NGF in the optic tectum has no effect on RGC axon morphology (Cohen-Cory and Fraser, 1995, Nature), thus NGF is a less likely candidate to be a stabilizing factor in this system.

Reviewer #3: Kutsarova et al. studied activity-dependent axonal refinement of retinal ganglion cells (RGC) in the optic tectum of albino Xenopus laevis tadpoles and analyzed the role of BDNF signaling pathway by suppressing TrkB, and P75NTR expression with morpholino oligonucleotide or quenching secreted BDNF with TrkB-Fc antibody. First, the authors used multiphoton live imaging combined with visual stimulation. They observed the few ipsilaterally projecting RGC axonal branch addition during uncorrelated visual activation is dependent on both p75 and TrkB but not BDNF. Then they found that BDNF suppresses branch elimination during correlated firing.

While the quantitative measures of axonal growth and retraction are attractive, the results are confusing and disjointed as ipsilateral axons are analyzed in Figures 1 and 2, but contralateral axons are used in Figure 3 (and Figure 4 is unclear). The distinct roles of TrkB and P75NTR are not well characterized. Furthermore, Discussion is full of conjectures and retrograde signaling for Stentian plasticity; the regulation of proBDNF and mBDNF and the local concentration of BDNF are discussed, but no data are presented. Also, the definition of Hebbian plasticity in this context is unclear whether the authors meant to argue LTP- or LTD-like phenomena.

We have attempted to provide a clearer justification for looking both at the ipsi axons, which can be considered a rare but serendipitous axon guidance error that provides a unique tool for studying how correlated firing affects axon branch behavior, as well as the contralateral projection which constitutes the vast majority, and in most animals the entirety, of the retinotectal projection at these stages. It is a fair criticism that we do engage in conjecture when constructing our final working model, most obviously with respect to the inferred actions of proBDNF and mBDNF which we did not test in this study, however, the elements in that model are all based on our findings interpreted in the light of the existing published literature.

We have made an effort to distinguish between Hebbian synaptic plasticity, such as LTP, and Hebbian structural plasticity but always applying modifiers like “structural” or “stabilization” when referring to axon dynamics driven by correlated firing.

*	Authors mention that "proBDNF signaling through presynaptic p75NTR is required for axonal retraction, whereas mBDNF signaling through presynaptic TrkB leads to axonal stabilization" (lines 67-68). If proBDNF-P75 is necessary for axonal retraction, why do the results in Figure 1 show that p75-MO prevents branch addition? How is it possible to reconcile? Does Stentian plasticity depend on both TrkB and P75NTR?

In the introduction (namely the sentence cited by the reviewer), we described studies by others in which presynaptic proBDNF-p75NTR signaling in the peripheral nervous system (neuromuscular junction) results in axonal retraction. Our data show that presynaptic p75NTR is required for increased growth in Stentian plasticity and also for suppression of branch loss during synchronous stimulation. Opposing functions of p75NTR in growth cone collapse and growth have previously been proposed to be due to the different p75NTR interactors expressed in the axon and/or its ligands. In these different scenarios p75NTR activation has been shown to either lead to RhoA activation or inhibition, as reviewed by Mathew et al., (2009). We hope that the revised text offers clearer context in the discussion.

*	"The Hebbian suppression of new branch additions…presumably the postsynaptic terminal, to induce Hebbian retrograde signals that inhibit the formation of new axonal branches" (line 113-123). The authors present discordant results of TrkB-Fc treated vs. neurotrophin receptor knock-down RGC axon and speculate retrograde signals from the postsynaptic terminal without evidence. How can such a retrograde signal differentiate ipsilateral axons from contralateral axons? Authors should provide supporting data.

From previous studies by our group and others, we know that postsynaptic NMDAR activation in response to correlated firing results in presynaptic RGC arbor stabilization (i.e. decreased branch initiation and loss (Rajan et al., 1999, Munz et al., 2014, Van Horn et al., 2017, Kesner et al., 2020). It has also been shown that expression of constitutively active CamKII in the postsynaptic tectal neurons results in RGC axon arbor stabilization, including suppression of new branch additions (Zou and Cline, 1996). Therefore, the literature provides irrefutable evidence for the existence of a putative retrograde signal. In our experiments, knock-down of either TrkB or p75NTR on the ipsi axon do not replicate the potent effect of sequestering BDNF with TrkB-Fc during synchronous stimulation, namely suppression of branch addition (Fig. 1F). Thus, the findings from the current study do not suggest that the retrograde signal leading to stabilization is BDNF. We discuss that BDNF acting postsynaptically is likely to induce the release or activation of other predicted retrograde signals whose molecular identity remains unknown. We believe that this is now clearer in the discussion and in the proposed model presented in Figure 5.

When considering ipsilateral axons, it is important to keep in mind that they constitute an insignificant fraction of retinotectal inputs in the Xenopus tadpole (approx. 1 in 5000). They are merely a useful tool that permits us to examine the interplay of patterned activity and molecular signaling, as we have the ability to control their firing patterns relative to the neighboring contralateral axons.

*	Data in Figure 3 is obtained from contralateral RGC axons (line 169) over four days. How does this relate to the data in Figures 1 and 2, which examined ipsilateral axons? Did the authors examine contra axonal refinement with the DAS system in Figures 1 and 2 and analyze ipsilateral axonal morphology over four days in Figure 3?

We have now added in the figure legends which data sets are contralateral or ipsilateral and attempted to provide a better rationale for the use of the ipsi axons in our study. To reiterate, the ipsi axons (which are not even present in the majority of animals) are most likely the result of pathfinding errors, but they offer a serendipitous and powerful tool for controlling visually evoked activity in a single, imageable axon while simultaneously stimulating the contralateral eye inputs. Our group has previously described the changes in morphology of ipsilaterally projecting RGC axon morphology in synchronous and asynchronous stimulation paradigms over days (Munz et al., *Science* 2014, Rahman et al. PNAS, 2020). This question is important because, as our data show correlated firing of neurons rather than just crude levels of activity, plays a key role in activity-dependent circuit refinement in development.

*	It is unclear whether Figure 4 analyzed ipsi- or contra-lateral axons. Another confusing aspect of the manuscript is that the authors go back and forth between ipsilateral and contralateral axon data and Stentian plasticity and Hebbian plasticity.

This was due to our poor labeling of the figure legends, which has now been corrected. We apologize for the oversight.

*	In lines 208-211, "From experiments where we electroporated p75-MO exclusively in the ipsi eye, we can conclude that p75NTR specifically on the asynchronously firing RGC axon mediates the response to Stentian signals that promote branch addition and extension." TrkB MO also has a similar effect, so it is still not conclusive, and more questions remain, such as the question of proBDNF vs. mBDNF or the identity of the putative retrograde signal.

The reviewer is correct that both p75-MO and TrkB-MO axons appear to lose the ability to differentially respond to asynchronous vs synchronous visual stimulation. Certain aspects of the dynamic branch remodeling do appear to be more clearly dependent on p75NTR than TrkB, as the p75-MO entirely abrogates any differences between the two stimulation paradigms. While we anticipated and would have been thrilled to have found that p75NTR and TrkB have no overlap in their actions on arbor remodeling, this is not what we observed.

*	In Figure 1C, how is the number of transient axonal terminals counted in the graph?

We have added this information to the new figure legend. We considered branches to be transient if they were both added and lost within the hour of stimulation represented in each trace. These are counted toward both the branch addition and branch loss quantifications.

*	In Figure 1 and G, the distribution of plots looks like there are two populations of response; one population that go up with asynchronous stimulation and then go down with synchronous stimulation, and the other population that declines over asynchronous and synchronous stimuli. In the former population, a few outliers appear to dictate the increase during asynchronous stimulation.

Because electroporation of MOs does not offer the possibility of targeting different classes of RGCs there is some heterogeneity of responses in our data. However, it is not the case that the effects we observed are dictated entirely by outliers. In an effort to exclude this possibility, we added 3 new control cells to the original 9 for this revision and the significant effects were still maintained (see the second figure in response to reviewer 2 above). Also, please note that we used non-parametric statistics which are far more resistant to large outliers for our analyses.

*	In Figure 1E, why does each curve not start from 1.0?

Figures 1D-E show the timecourse of the rates of branch addition and loss normalized to the rate during baseline collection in darkness. Since each point represents an average of 20 min rather than an hour as plotted in the bar graphs, the initial value may differ from 1.0. but the average over the darkness period will be 1.0. We have now updated the figure legend for clarity. Thank you for raising this question.

Once again I would like to take this opportunity to thank the reviewers for their logical and thoughtful reading of our manuscript which has resulted in a number of excellent suggestions for improvement. We hope you will now consider that the paper is innovative and exciting and also now meets the standards for publication in its current form. Thanks on behalf of our team..
